# Using laboratory data to categorise CD4 laboratory turn-around-time performance across a national programme

**DOI:** 10.4102/ajlm.v7i1.665

**Published:** 2018-06-28

**Authors:** Lindi-Marie Coetzee, Naseem Cassim, Deborah K. Glencross

**Affiliations:** 1 National Health Laboratory Service, National Priority Programme, Johannesburg, South Africa; 2 Department of Molecular Medicine and Haematology, University of the Witwatersrand, Johannesburg, South Africa

## Abstract

**Background and objective:**

The National Health Laboratory Service provides CD4 testing through an integrated tiered service delivery model with a target laboratory turn-around time (TAT) of 48 h. Mean TAT provides insight into national CD4 laboratory performance. However, it is not sensitive enough to identify inefficiencies of outlying laboratories or predict the percentage of samples meeting the TAT target. The aim of this study was to describe the use of the median, 75th percentile and percentage within target of laboratory TAT data to categorise laboratory performance.

**Methods:**

Retrospective CD4 laboratory data for 2015–2016 fiscal year were extracted from the corporate data warehouse. The laboratory TAT distribution and percentage of samples within the 48 h target were assessed. A scatter plot was used to categorise laboratory performance into four quadrants using both the percentage within target and 75th percentile TAT. The laboratory performance was labelled good, satisfactory or poor.

**Results:**

TAT data reported a positive skew with a mode of 13 h and a median of 17 h and 75th percentile of 25 h. Overall, 93.2% of CD4 samples had a laboratory TAT of less than 48 h. 48 out of 52 laboratories reported good TAT performance, i.e. percentage within target > 85% and 75th percentile ≤ 48 h, with two categorised as satisfactory (one parameter met), and two as poor performing laboratories (failed both parameters).

**Conclusion:**

This study demonstrated the feasibility of utilising laboratory data to categorise laboratory performance. Using the quadrant approach for TAT data, laboratories that need interventions can be highlighted for root cause analysis assessment.

## Introduction

The National Health Laboratory Service (NHLS) provides CD4 testing for staging HIV-positive persons and monitoring for people living with HIV on antiretroviral therapy (ART) within the public health sector in South Africa. CD4 testing is currently offered at 52 laboratories using Beckman Coulter equipment and the PanLeucogating method.^1,2^ While most CD4 testing takes place at district and regional hospitals, samples predominantly originate from primary healthcare facilities.^3^

An integrated tiered service delivery model for CD4 testing has been implemented within the NHLS in South Africa^3^ to facilitate widespread universal coverage of CD4 services across the CD4 laboratories. The model strives to provide CD4 testing technology that appropriately matches service delivery requirements,^3^ based on the daily test volumes in any given laboratory and the laboratory’s respective demarcated service coverage precinct.^3^ Five testing tiers are defined for CD4 services ranging from Tier-5 (high volume, centralised testing sites) to Tier-1 (point-of-care sites used to extend laboratory services into hard-to-reach areas).^3^ Most CD4 laboratories process more than 100 samples per day, with some high volume sites processing in excess of 500 samples per day.^1,3^

To ensure that all CD4 results are received in a timely fashion and expedite local antiretroviral (ART) treatment according to guidelines at the clinical interface, CD4 laboratories are mandated to report all CD4 results within a total laboratory turn-around time (TAT) of 48 h. The South African 2015 ART guidelines^4^ prescribe a six-month CD4 testing schedule for individuals diagnosed with HIV infection but not eligible for ART.^4^ Despite adjustment of these guidelines in 2016 to incorporate a universal ‘test and treat’ strategy, where all HIV-positive persons are eligible for treatment irrespective of their CD4 counts, the CD4 guideline^5^ testing and treatment requirements have been retained. Emphasis is put on baseline CD4 testing for assessing immune status and fast tracking individuals into care, especially < 200 cells/ul, or screening for opportunistic infection.^6,7^ Adults on ART thus undergo CD4 testing at 12 months and then annually when indicated clinically.^4^ The overall mandate for care is to ensure timely ART initiation for all eligible persons within two weeks of CD4 testing and within seven days for fast tracking.^4,5^

Mean TAT has been used to report service efficiency and laboratory performance.^8,9^ The distribution of mean laboratory TAT is typically a non-Gaussian distribution;^10^ use of mean TAT is thus not an accurate reflection of overall TAT performance in laboratories. Hawkins describes TAT distributions as demonstrating a positive skew typically to the right, highlighting the need to assess tail size.^10^ Therefore, to better understand TAT performance, an analysis of median TAT is a more useful measure to provide information about the performance norm, such as the median performance of laboratories. The analysis also provides information about the exception to the median, that is, the outliers (the tail size),^11^ allowing for a more balanced assessment of TAT performance and removal of attention on a single indicator: mean or median TAT.

CD4 results need to be available to meet the seven- to 14-day standard of care for ART services. The aim of this study was to investigate a method to highlight TAT performance to close the gap on service deficiencies. CD4 TAT efficiencies were assessed using the median and 75th percentile TAT, as well as the percentage of samples within the TAT target of 48 h.

## Methods

### Ethical considerations

Ethics clearance for this work was obtained from the University of the Witwatersrand (study approval number: M1706108).

### Turn-around time data extraction

Retrospective data were extracted from the corporate data warehouse for the 2015–2016 fiscal year (01 April 2015 to 31 March 2016). Fields included the episode number, registered date, reviewed date, TAT in hours, flow cytometer serial number and the name of the CD4 laboratory. Both the registered and reviewed dates are generated automatically by the laboratory information management system (LIMS) and used to calculate the TAT. TAT data were analysed using Microsoft Access 2013 and Excel 2013 (Albuquerque, New Mexico, United States) and Stata, version 12 (College Station, Texas, United States). Data anomalies such as unreviewed CD4 results, samples reviewed before they had been registered due to incorrect date and time settings, for example were removed using Stata.

**FIGURE 1:**
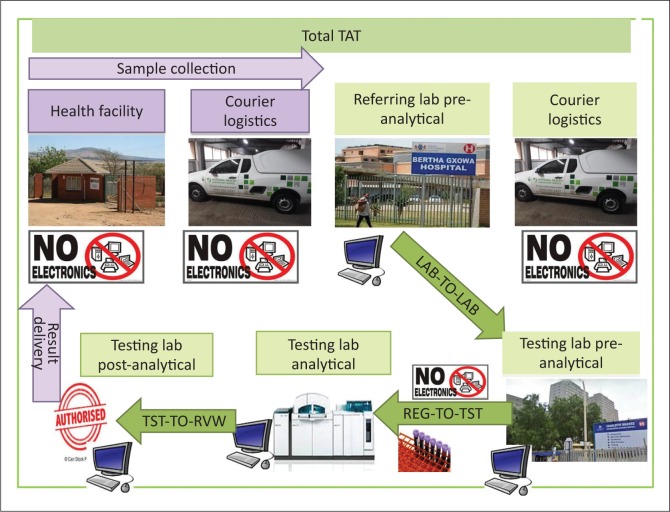
A logical flow of samples from the referring to the testing laboratory, indicating the individual components of total laboratory turn-around time (green). This consists of LAB-TO-LAB (time from registration at referral laboratory to registration at testing laboratory); REG-TO-TST (time to capture CD4 result from registration at testing laboratory) and TST-TO-RVW (time from CD4 result to authorisation on LIMS). Purple elements represent sample collection at the health facility and transport to the referring laboratory, as well as result delivery back to the health facility, which are not included in the laboratory turn-around time calculations.

The CD4 laboratory TAT was calculated from registration on the LIMS at the referring laboratory to results reviewed or authorised by a senior medical technologist at a CD4 testing laboratory (Figure 1). The CD4 TAT measure was used in this study as an indicator of the overall efficiency of the laboratory network to transport CD4 samples between laboratories and conduct testing. Although it would be optimal to assess facility-to-facility TAT, this was not possible as an adequate sample tracking system is not in place.

### Turn-around time distribution analysis

GraphPad Prism version 6 software (La Jolla, California, United States) was used to assess TAT distribution on a histogram indicating the 25th, 50th, 75th, 95th and 99th percentiles across all CD4 samples tested within the 2015–2016 period. Stata was used to generate descriptive statistics, including the mean, median, inter-quartile range and percentiles.

### National turn-around time range distribution analysis

Specimen-level laboratory TAT data (hours) of all 52 CD4 testing laboratories were analysed and categorised as satisfactory (≤ 48 h) or unsatisfactory (> 48 h) using Stata software. The annual performance plan target is for 85% of samples to meet the 48 h TAT target.^8^

### Individual laboratory turn-around time performance categorisation

A scatter plot was created using Microsoft Excel reporting the respective laboratory’s percentage of samples within the target TAT (x-axis) and the 75th percentile TAT (y-axis) to categorise individual laboratory performance into four quadrants. Laboratories in Quadrant 1 (Q1) had both ≥ 85% of samples within the target TAT and a 75th percentile TAT ≤ 48 h (both parameters within target), and were categorised as having ‘good’ performance. Laboratories in Quadrant 2 (Q2) were defined as laboratories with ≥ 85% of samples within the target TAT but a 75th percentile TAT is > 48 h (only the 75th percentile did not meet the target), and were categorised as having ‘satisfactory’ performance. Laboratories in Quadrant 3 (Q3) had < 85% of samples within the target TAT and a 75th percentile TAT > 48 h (both parameters out of target), and were categorised as having ‘poor’ performance. Finally, laboratories in Quadrant 4 (Q4) had < 85% of samples within the target TAT (out of target) and a 75th percentile TAT ≤ 48 h (within target), and were categorised as having ‘satisfactory’ performance. Quadrants were colour coded and labelled.

### Analysis of contributing factors for turn-around time performance

All laboratories from Q1 with good performance were selected for workflow analysis and TAT component analysis. In addition, two laboratories with satisfactory performance (Q4) and two laboratories categorised with poor performance (Q3) were assessed for workflow and laboratory TAT components. TAT components included: (1) laboratory-to-laboratory: time from registration at the referral laboratory to registration at the CD4 testing laboratory; (2) registration-to-testing: time from registration at the testing laboratory to capture of the CD4 result on LIMS; and (3) testing-to-review: time from capture of CD4 result on LIMS to authorisation (review and confirmation of physical result printout from flow cytometer platform) by a senior medical technologist (Figure 1). For the workflow analysis, the hour of the day (1–24) when samples were registered, tested and reviewed were analysed to assess hourly frequencies to determine the synchronisation of these laboratory activities. The data were analysed and reported using Microsoft Excel, GraphPad Prism and Stata, with both the median and 75th percentile components of the TAT reported. Pre-analytical (facility to referring laboratory) and post-analytical (review to result delivery to a service provider) times were outside the scope of this study.

## Results

The extracted data contained 3 618 856 rows of TAT data for the 2015–2016 fiscal year. Exclusion of data anomalies removed 3.6% (131 629 rows) of data. Data anomalies included invalid dates, for example ‘1800/01/01’ caused by an incorrect computer clock and TAT calculation errors. The final TAT analysis was conducted on a dataset of 3 487 227 CD4 samples tested across the NHLS network between April 2015 and March 2016. For the period reported, the mean TAT was 22 h, with a median of 17 h and an inter-quartile range of 11 h–25 h.

### National turn-around time distribution

**FIGURE 2:**
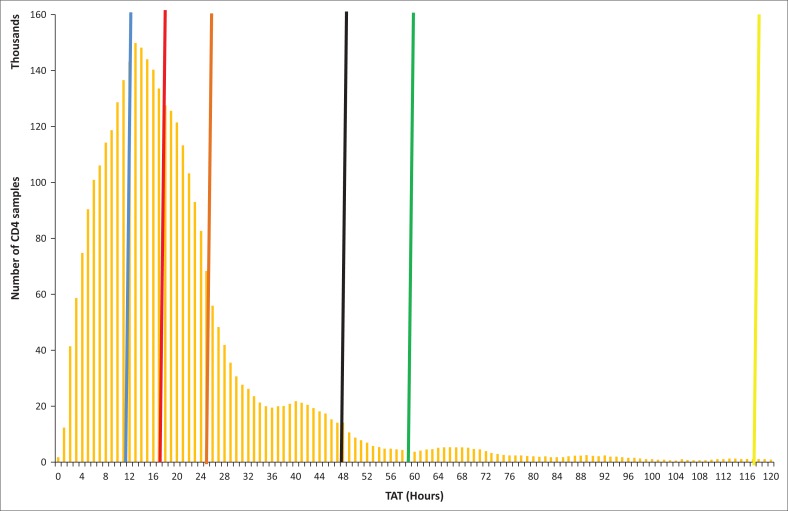
Histogram displaying turn-around time distribution across a national CD4 programme with the blue, red, orange, green and yellow lines representing the 25th, 50th (median), 75th, 95th and 99th percentiles, respectively, and the bold black line indicating the target turn-around time of 48 h.

The 2015–2016 fiscal year CD4 TAT data was skewed to the right with a mode of 13 h. Figure 2 depicts a right-tailed distribution with a long tail resulting in a skewness of 4.6. A 25th percentile TAT of 11 h and a median of 17 h were reported, both within the 48 h target. A 75th percentile TAT of 25 h was reported. The TAT target of 48 h was exceeded at the 95th (58 h) and 99th percentile (117 h), representing 6.8% of all samples tested.

### National turn-around time range distribution

**FIGURE 3:**
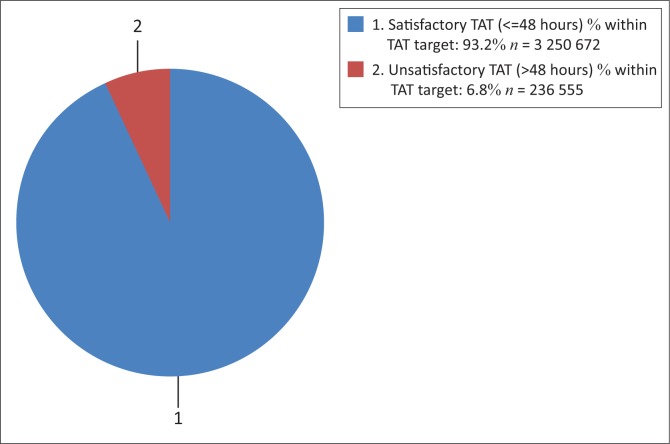
A pie graph representing sample volumes and percentage of samples with satisfactory (≤ 48 h) and unsatisfactory (> 48 h) CD4 turn-around time performance in the 2015–2016 fiscal period overall.

Figure 3 reveals that 93.2% of CD4 samples had a TAT ≤ 48 h, that is satisfactory TAT. There were 236 555 (6.8%) samples with a TAT > 48 h, meaning unsatisfactory TAT.

### Individual laboratory turn-around time performance

**FIGURE 4:**
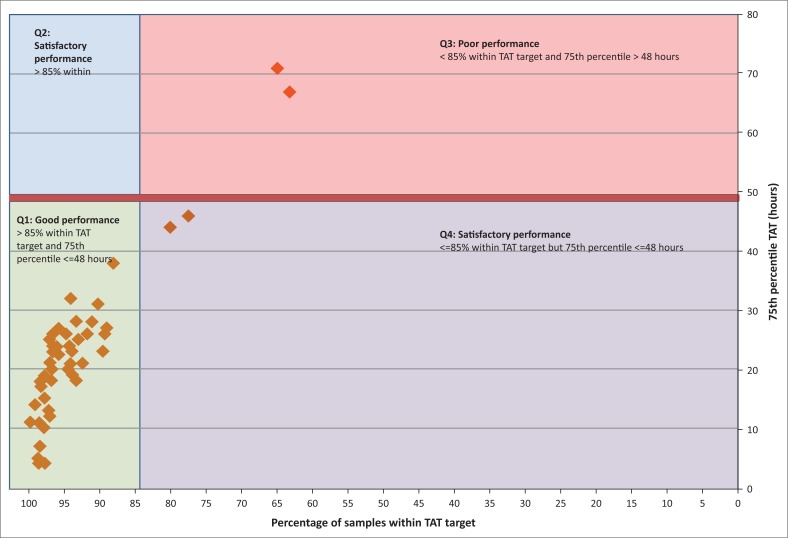
Scatter plot of CD4 laboratories reporting the percentage of samples within the TAT target (x-axis) and the 75th percentile (y-axis) with TAT performance categorised into four quadrants (*n* = 52). Quadrant 1 (Q1, green) represents good performance with both > 85% of samples within the TAT target and the 75th percentile < 48 h. Quadrant 2 (Q2, blue) identifies satisfactory performance with > 85% of samples within the TAT target, but with a long tail at the 75th percentile (> 48 h). Quadrant 3 (Q3, red) reports poor performance where both the percentage of samples within the TAT target and the 75th percentile values are not meeting their targets. Quadrant 4 (Q4, purple) represents satisfactory laboratories with < 85% of samples within the TAT target, but within the 75th percentile target of 48 h.

Analysis of individual laboratory TAT performance (Figure 4) showed that 48/52 (93.2%) testing laboratories were represented in Q1, meeting both the 85% of samples within the target TAT and a 75th percentile target ≤ 48 h. Overall, 50 out of 52 (96.2%) laboratories achieved the 75th percentile target of a ≤ 48 h TAT (Q1 and Q4). There were no representative laboratories in Q2 (satisfactory performance as defined in the methodology), and Q4 had two laboratories with satisfactory performance. There were two laboratories in Q3 that showed poor performance, with a < 85% of samples within the target TAT and a 75th percentile TAT of > 48 h. These laboratories reported 63% and 65% of samples with the target TAT, and their 75th percentile values were 67 h and 71 h, respectively.

Laboratories represented in Q1 as having good performance included 31 using the high-throughput fully-automated MPL/CellMek testing platform and 17 using the low-throughput automated Aquios testing platform with varying test volumes. No correlation between test volumes and TAT was indicated in this study (data not shown, *p* > 0.1). Platform and test volumes for Q3 and Q4 also included both Aquios and MPL systems with low to high test volumes.

### Analysis of contributing factors for turn-around time performance

**FIGURE 5:**
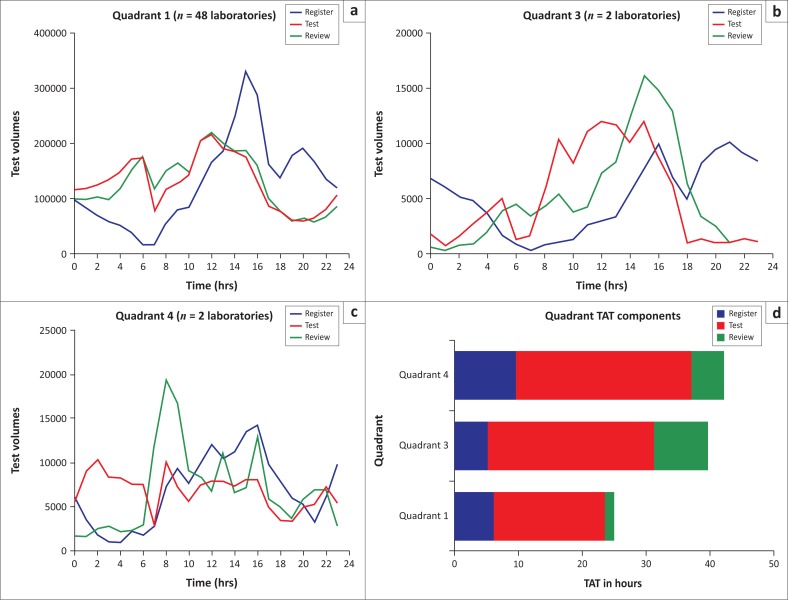
(a) Represents the workflow over a 24 h period of all good performing laboratories (*n* = 48) versus, (b) a satisfactory performing laboratory (as identified in Figure 4, Q4), (c) Represents two poor performing laboratories, (d) Indicates the breakdown of TAT into registration time in green, testing time in red and review time in blue.

Workflow analysis was done for data of all laboratories by quadrant. Among laboratories with good performance (Q1; *n* = 48), sample testing and review was done throughout the day, with a decline in testing and reviewing from 16:00 to midnight (Figure 5a). Registration peaked between 12:00 and 16:00, with another peak at 20:00–22:00. Testing and review followed each other closely during the 24 h testing period, meaning that there was no significant time lapse between testing and review.

Among laboratories with poor performance (Q3; *n* = 2), registration peaked around 15:00–16:00 and again at 20:00–22:00, with testing occurring between 07:00 and 18:00 (daytime) (Figure 5b). A significant lapse was noted between testing and review, with a peak in review at 14:00–18:00.

The two laboratories with satisfactory performance (Q4) showed testing throughout the 24 h, with review following testing during the day, peaking at 06:00–10:00, with very little reviewing taking place between midnight and 06:00 (Figure 5c).

When comparing TAT components for the three quadrants, registration took the longest for Q4 laboratories (9 h). Testing times were about equal for Q4 and Q3 (25 h–27 h), but were significantly longer than testing time for Q1 (17 h) (Figure 5d). Review times were also significantly longer for both Q3 (5 h) and Q4 (8 h) compared with Q1 (1.3 h on average).

## Discussion

Turn-around time is one of the most perceptible aspects noted by clinicians with respect to laboratory services^10,12^ and is frequently used to gauge laboratory service performance.^9^ It can also be used by the laboratory service provider to appraise efficiencies across a network of testing facilities. Laboratory test TAT depends on the nature of test, the clinical significance and time to clinical intervention.^10,12^ For example for CD4 services, determining an acceptable local TAT target is based on the standard of care for enrolling patients onto ART. In South Africa, this equates to ART initiation within 14 days of CD4 testing or seven days where a CD4 is < 200 cells/µL.^4,5^ Using these guidelines, national and individual laboratory CD4 TAT performance was assessed against the target of 48 h.

Currently within the NHLS, the average or mean TAT of non-normally distributed data is routinely reported to assess the performance of CD4 laboratories. This study, however, indicated that the median TAT is a better measure of central tendency, given the right tailed distribution. In addition to using the median, two more measures, namely the 75th percentile and percentage of samples within the target TAT, were introduced to assess tail size and the overall proportion of samples meeting the 48 h TAT target. The intersection of these three variables provide a more accurate assessment of CD4 TAT performance, as laboratories can report a median and 75th percentile within TAT, but fail to meet the 85% threshold requirement for samples within the target TAT.

For the study period overall, 93.2% of samples were reported within the NHLS 48 h TAT target. The median or 50th percentile CD4 TAT of 17 h reported here further revealed that a large number of samples were reported in less than a third of the time of the TAT target. However, a small proportion of samples failed to meet the target TAT. The national CD4 TAT distribution demonstrated a long tail to the right. This may be due in part to local TAT challenges causing delays, as reflected at the 95th TAT percentile of 58 h. Even at the 99th percentile, CD4 results were released within 5 days (117 h); that should be well within the local National Department of Health guidelines for ART treatment initiation standard of care.^4,5^ Time delays from facility to referral laboratory and review to result in hand were not included, as these times are not currently captured.

Plotting the percentage of samples within the target TAT versus the 75th percentile as a scatter plot provides important visual insights about how laboratories perform in relation to their peers and against set targets. This information can potentially be incorporated into a dashboard with stop lighting for individual laboratories, or regions for management interventions to understand at a glance, and to hone in on sites at risk for bottlenecks in service delivery.

Good overall laboratory TAT performance was noted for 48 out of 52 laboratories, only two laboratories had satisfactory performance and only two had poor performance (both percentage of samples within target and 75th percentile ≤ 48 h not met). It is to be expected that centralised testing will result in longer pre-analytical laboratory-to-laboratory TAT as samples are brought into the testing facility from multiple distant referral sites (Figure 1). Differences in performance (quadrant placements) were, however, not related to the location of laboratories, testing platform, test volumes, staff component or hours worked (i.e. 24 h service vs 8 or 12 h).

TAT is affected by a multitude of factors or combinations thereof, that may or may not be related to test volumes. These may include synchronisation of registration, testing and review activities, pre-analytical delays, frequency of courier logistics, instrument capacity and downtime, staffing availability and competency, LIMS bandwidth and optimal workflow.

The deficiency of the current LIMS system in use in the NHLS is that it does not offer detailed end-to-end tracking capability to identify all components of pre-analytical and analytical TAT. Auto review, using delta check rules, to automatically release CD4 results is currently not in use and may dramatically reduce testing-to-review TAT. Total laboratory automation (for larger core laboratories) and auto verification showed a marked decrease in TAT for various laboratory tests.^13,14^ In the absence of a fully optimised LIMS system, on-site audits are indicated for laboratories performing outside the acceptable target, to at least ensure that the laboratory testing or reviewing phase is optimised. Audits as a tool for continued improvement of laboratory TAT was recently reported for chemistry results in a typical South African teaching hospital, confirming its application in TAT root cause analysis of outlying performers.^15^ In addition, a system for long-term monitoring of performance should also be in place to ensure continued acceptable performance by all testing laboratories.

The workflow analysis of laboratories depicted in Figure 5 showed the impact of delayed testing and reviewing on meeting TAT targets. The main difference between laboratories was workflow synchronisation as evidenced by the time lapse between sample registration and testing, and testing and reviewing. Laboratories with poor performance were assisted through on-site visits to assist with workflow. Root cause analysis identified that the unavailability of qualified staff may lead to bottlenecks in testing or authorisation.

The desktop analysis of TAT described in this article shows that it is possible to categorise TAT performance and identify the in-laboratory component contributing to delays in TAT.

### Limitations

This study used predominantly data from the corporate data warehouse to assess laboratory TAT data. The data presented here cannot differentiate all the various components of pre-analytical, analytical and post-analytical delays for CD4 testing TAT. Time delays from facility to referral laboratory and review to result in hand are not currently monitored, so in the absence of a local sample tracking system, it is not possible to assess end-to-end CD4 service delivery.

### Recommendations

The following recommendations are proposed to improve TAT monitoring:

- Develop a weekly TAT monitoring report incorporating the aspects described above namely, scatter plot, component analysis and, where required, workflow analysis.
- Develop an operational corporate data warehouse TAT dashboard to facilitate real-time access to TAT data.

### Conclusion

This study demonstrated the feasibility of establishing laboratory TAT performance using secondary data from the corporate data warehouse. Additionally, the shortcomings of using only a mean or median to assess TAT performance are highlighted. Once a monitoring system has been developed to provide real-time tail size data, the national CD4 programme would be able to plan focused interventions to proactively resolve poor service delivery levels.
